# PReS-FINAL-2187: Use of screening tests in patients presenting to paediatric rheumatology with suspected haemophagocytic lymphohistiocytosis/macrophage activation syndrome

**DOI:** 10.1186/1546-0096-11-S2-O22

**Published:** 2013-12-05

**Authors:** M Cruikshank, P Anoop, O Nikolajeva, A Rao, K Rao, K Gilmour, D Eleftheriou, PA Brogan

**Affiliations:** 1 Rheumatology, Great Ormond Street Hospital, London, UK; 2 Haematology, Great Ormond Street Hospital, London, UK; 3 Bone Marrow Transplantation, Great Ormond Street Hospital, London, UK; 4 Immunology, Great Ormond Street Hospital, London, UK

## Introduction

Haemophagocytic lymphohistiocytosis (HLH) is a severe condition in which there is extreme uncontrolled inflammation, and may progress rapidly to multi-organ failure and death. HLH may be genetic (primary HLH), or secondary to infection or autoimmune/ autoinflammatory conditions; if the latter, it is also referred to as macrophage activation syndrome (MAS). Distinguishing between primary HLH and MAS is challenging but important since the former requires different therapeutic approaches including allogeneic haematopoietic stem cell transplantation (HSCT) for long-term survival. HLH screening tests are now being used in patients presenting with suspected MAS. In systemic Juvenile Idiopathic Arthritis (sJIA), some patients demonstrate temporary perforin expression abnormalities that resolve with disease control. The utility of other screening tests in a rheumatology context is unknown.

## Objectives

The purpose of this study was to describe the performance of screening tests used in the HLH/MAS work up of children presenting to a specialist paediatric rheumatology centre, and review outcomes of those with screening abnormalities.

## Methods

A database exists of patients who had screening tests for suspected HLH/MAS. Screening tests (flow cytometry) included: intracellular expression of perforin in CD56+ Natural Killer (NK) cells; CD107a Granule Release Assay (GRA) in response to PHA in NK cells or anti-CD3 stimulation of CD8 lymphocytes; in male patients Signal Lymphocyte Activating Molecule Associated Protein (SAP, associated with XLP1) and X-linked Inhibitor of Apoptosis Protein (XIAP, associated with XLP2) expression. All tests requested by paediatric rheumatology over a 5 year period (2007-2011) were included. Patient records and laboratory parameters were retrospectively reviewed.

## Results

22 patients (15 female), median age 6.5 years (range 0.6-16) underwent screening tests, with median follow-up of 16 months (range 3-51). At presentation only 2/22 (9%) clinically met HLH criteria. Screening results were available for 20 patients; 7 (35%) had at least one persistent abnormality in any one of the tests; this group was associated with 57% mortality or need for HSCT, compared to 8% with no abnormality on any of the tests (p = 0.03). 6/20 (30%) had persistently abnormal GRA: their final diagnoses were sJIA with MAS (n = 3); primary HLH (n = 2); and overlap syndrome (n = 1). 1/4 boys screened for XIAP had absent expression with subsequent genetic confirmation of XLP2. 18 patients had perforin screened, and 5 boys were screened for SAP expression; all had normal results.

## Conclusion

Primary HLH and MAS may overlap clinically and screening in patients with suspected MAS is warranted; overall 14% had an eventual diagnosis of primary HLH. Persistently abnormal GRA defines a high risk group with poor outcome (mortality or need for HSCT) possibly due to an unidentified HLH gene. The effect of immunosuppression on the GRA was not assessed. Further research is required in those with abnormal GRA to help understand the pathogenesis of HLH/MAS.

## Disclosure of interest

None declared.

